# Characterization of *NS5A* and *NS5B* Resistance-Associated Substitutions from Genotype 1 Hepatitis C Virus Infected Patients in a Portuguese Cohort

**DOI:** 10.3390/v10050223

**Published:** 2018-04-26

**Authors:** Ruben Brandão, Rute Marcelino, Fátima Gonçalves, Isabel Diogo, Ana Carvalho, Joaquim Cabanas, Inês Costa, Pedro Brogueira, Fernando Ventura, Ana Miranda, Kamal Mansinho, Perpétua Gomes

**Affiliations:** 1 Molecular Biology Laboratory, LMCBM, SPC, HEM—Centro Hospitalar Lisboa Ocidental, 1349-019 Lisboa, Portugal; mfmgoncalves@chlo.min-saude.pt (F.G.); ifmadeira@chlo.min-saude.pt (I.D.); pkarvalho@iol.pt (A.C.); jcabanas65@gmail.com (J.C.); icosta@chlo.min-saude.pt (I.C.); gomes.perpetua@gmail.com (P.G.); 2 Global Health and Tropical Medicine (GHTM), Unit of Medical Microbiology, Instituto de Higiene e Medicina Tropical (IHMT), Universidade NOVA de Lisboa (UNL), 1349-008 Lisboa, Portugal; rute.marcelino@ihmt.unl.pt; 3 Serviço de Doenças Infeciosas, HEM—Centro Hospitalar Lisboa Ocidental, 1349-019 Lisboa, Portugal; pbrogueira@gmail.com (P.B.); venturafa@gmail.com (F.V.); anacbcm@gmail.com (A.M.); mansinho.k@gmail.com (K.M.); 4 Centro de Investigação Interdisciplinar Egas Moniz, CiiEM, ISCSEM, 2829-511 Almada, Portugal

**Keywords:** hepatitis C virus, direct-acting antivirals, resistance-associated substitutions, *NS5A*, *NS5B*

## Abstract

This study is focused on the prevalent NS5 coding region resistance-associated substitutions (RASs) in DAA-naive genotype (GT)1 HCV-infected patients and their potential impact on success rates. Plasma RNA from 81 GT1 HCV-infected patients was extracted prior to an in-house nested RT-PCR of the NS5 coding region, which is followed by Sanger population sequencing. *NS5A* RASs were present in 28.4% (23/81) of all GT1-infected patients with 9.9% (8/81) having the Y93C/H mutation. *NS5B* RASs showed a prevalence of 14.8% (12/81) and were only detected in GT1b. Overall 38.3% (31/81) of all GT1 HCV-infected patients presented baseline RASs. The obtained data supports the usefulness of resistance testing prior to treatment since a statistically significant association was found between treatment failure and the baseline presence of specific NS5 RASs known as Y93C/H (*p* = 0.04).

## 1. Introduction

**Figure 1:**
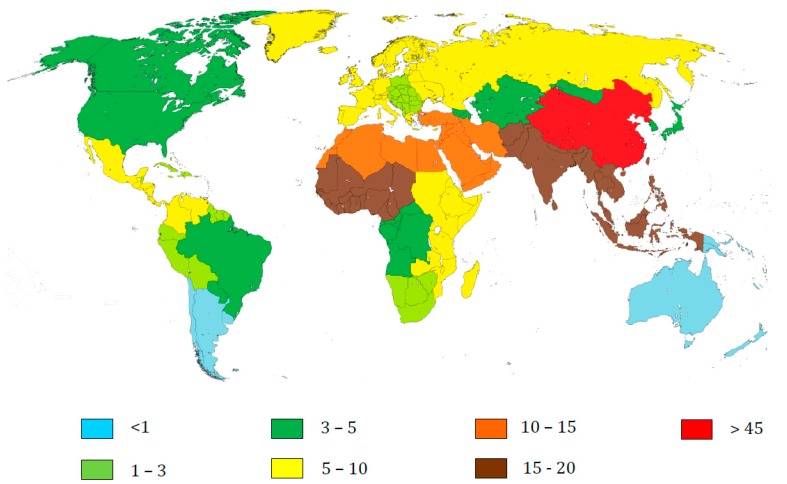
Worldwide estimated people with anti-HCV antibodies in 2016 (in millions) in 21 GBD regions. Data obtained from References [1,3].

The hepatitis C virus (HCV) is considered to be the second leading cause of hepatocellular carcinoma (HCC) and further co-morbidities such as cirrhosis and other chronic liver diseases. Data from 2017 on the Global Burden of Disease Study (GBD) estimate that about 158 million people are seropositive for HCV (see Figure 1) [1]. Moreover, the number of HCV-associated mortalities has also seen a decrease over the years to about 489,000 reported deaths of which more than 159,500 were related to liver cancer due to HCV [2].

HCV is highly diversified and demonstrates a geographic distribution since it is currently divided into seven genotypes and more than 80 known subtypes of which the first genotype is the most prevalent. The first genotype globally accounts for 83.4 million (46.2%) of all HCV infections while HCV GT3 constitutes the second most common with 54.3 million (30.1%) cases worldwide [4].

Since 2014, IFN-free regimens constitute the best treatment options in chronic Hepatitis C infection for treatment-naive (TN) and treatment-experienced (TE) patients with compensated or decompensated liver disease as a result of the ease of use through all-oral DAAs, increased tolerability, and the dramatic increase in viral suppression demonstrating SVR rates superior to 90% [5,6,7,8,9,10,11,12,13,14,15,16,17,18,19,20]. Current *NS5A* inhibitors such as ledipasvir (LDV) and velpatasvir (VEL) exhibit a high genetic barrier to resistance when compared to the first generation precedents. However, this is not as high as the nucleoside inhibitors (NIs) of the *NS5B* polymerase currently known as sofosbuvir [21]. However, DAAs regimens are still not 100% effective due to low adherence to treatment as well as due to the presence of baseline RASs, which show that only high risk *NS5A* and *NS5B* RASs appear to have an impact on the response to treatment when exposing an HCV-infected patient to an IFN-free therapy based in SOF/LDV ± RBV for 12 weeks [22].

Recent studies have described a prevalence of baseline RASs in HCV-infected patients that can range from 1 to 80% [23], which leads to a decrease in SVR rates between 1% and 50% [23]. *NS5A* constitutes the most important genomic region considered for the screening of associated clinically relevant RASs since it displays the highest number of mutations in GT1 HCV while regimens based on *NS5B* polymerase inhibitors, particularly NIs, tend to exhibit a low prevalence of baseline RASs [24] and the S282T mutation, although very relevant in therapeutic failures after a treatment with NIs, is rarely detected at baseline [25]. Furthermore, the ION clinical trials [15,16,17] have shown that reduced rates of SVR observed after a regimen of SOF + LDV for 12 weeks in patients infected with GT1 HCV were essentially associated with the presence of baseline *NS5A* RAS, which conferred an in vitro high fold resistance (>100) to ledipasvir [26].

Our main goal was to study the profile of NS5 coding region RASs in DAA-naive genotype 1 among HCV-infected patients. Additionally, three separate hypotheses were tested to ascertain an association between treatment failure and: (a) the non-discriminatory presence of baseline NS5 RASs, (b) the baseline presence of *NS5A*, *NS5B* and/or *NS5A* + *NS5B* class RASs, and (c) the baseline presence of specific NS5 RASs.

## 2. Materials and Methods

### 2.1. Samples

This study was approved on 6 September 2017 by the Ethical Committee for Health of Centro Hospitalar de Lisboa Ocidental (CHLO) with the RNEC Registry Number: 20170700050.

A group of 81 clinical samples of plasma from DAA-naïve GT1a and GT1b-infected patients selected to initiate treatment with DAAs between 2015 and 2017 were analyzed for baseline NS5 RASs profiling. The viral load had been previously determined using the Quantitative/Qualitative COBAS^®^ AmpliPrep/TaqMan^®^ HCV Test v2.0 from Roche Molecular Diagnostics (Basel, Switzerland). The HCV genotype had been previously determined using the VERSANT^®^ HCV Genotype 2.0 Assay Line Probe Assay (LiPA) from INNOGENETICS/Siemens Healthineers (Ghent, Belgium). Twenty-five samples corresponded to GT1b (31%) while GT1a was the predominant subtype accounting for 56 HCV infected patients (69%). Moreover, 3 out of the 81 patients had failed a previous treatment with DAAs (4%), and two patients showed an unknown treatment outcome status (2%) due to lack of adherence to the associated regimen or loss to follow-up evaluation before the SVR12 visit, which resulted overall in an SVR rate of 94% (76/81) (see Table 1).

### 2.2. HCV RNA Extraction

HCV nucleic acids were extracted from 500 µL of plasma previously conserved at −80 °C, which ultimately eluted 45 µL of RNA product using the bioMérieux’s automated NucliSENS^®^ EasyMAG^®^ system v2.0 (with silica, Boxtel, The Netherlands), according to the manufacturer’s protocol. 

### 2.3. PCR and Sequencing Primers Design

In order to cover the *NS5A* and *NS5B* coding regions, each amplification reaction used two primers including one forward primer and one reverse, which encompasses a length of approximately 4800 bp and 3700 bp for the RT-PCR (outer PCR) and nested PCR (inner PCR), respectively (see Table S1). A total set of 10 primers (Table S1) of which two were subtype specific were selected to cover the NS5 coding region of GT1 HCV. However, since the 3′-end of the *NS5B* gene is a poly-U (U/C) region highly composed of hairpins and secondary structures that obstruct the annealing process, the associated FW6 sequencing primer was unable to completely hybridize during the sequencing reactions. Three important amino acid positions (A553, G554, and S556) associated with resistance to dasabuvir were consequently left uncovered. Primers were synthesized by Thermo Fisher Scientific (Waltham, MA, USA) and TIB MOLBIOL (Berlin, Germany). 

### 2.4. cDNA Synthesis and Nested-PCR

HCV RNA was reverse transcribed using the OneStep RT-PCR Kit from QIAGEN (Hilden, Germany) and this first round of amplification (outer PCR) was performed under the following final conditions: OneStep RT-PCR Buffer (1×), 2.5 µL of OneStep RT-PCR Enzyme Mix, dNTPs Mix (400 µM each), 0.5 µM sense primer FW1, 0.5 µM anti-sense primer RV1, 10 U/reaction of protector RNase inhibitor, 10 µL of template RNA, and RNase-Free water to make up a final reaction volume of 50 µL. Cycling conditions were as follows: a reverse transcription step at 45 °C for 30 min, a HotStarTaq polymerase activation step at 95 °C for 15 min to initiate PCR, a touchdown PCR step of 16 cycles of 10 s at 94 °C (denaturation step), 45 s at 63 °C with −1 °C/cycle (annealing step), and 5 min at 68 °C (extension step), a final PCR step of 30 cycles of 10 s at 94 °C, 45 s at 48 °C, and 5 min at 68 °C with a time increment of +3 s/cycle, and a final elongation step of 10 min at 68 °C. The reaction was then cooled down to 4 °C and RT-PCR products were stored at −20 °C or immediately used in the nested PCR step.

Two microliters of the RT-PCR reaction product were amplified in a second in-house nested PCR (inner PCR) divided in two mixes, which was as recommended by the Expand High Fidelity PCR System Kit (Roche), under the following conditions: Mix 1 (dNTPs mix (200 µM each), 0.3 µM sense primer FW2, 0.3 µM anti-sense primer RV2, and RNase-Free water to make up a mix volume of 23 µL) and Mix 2 (Expand High Fidelity Buffer (1×) without MgCl_2_, 0.4% DMSO, 3 mM MgCl_2_, 3U/reaction of Expand High Fidelity Enzyme mix, and RNase-Free water to make up a mix volume of 25 µL). Lastly, 2 µL of template cDNA were mixed with 23 µL of Mix 1, which is followed by adding 25 µL of Mix 2 to make a final reaction volume of 50 µL for each sample. Cycling conditions include a denaturation step at 95 °C for 3 min, a touchdown PCR step of 16 cycles of 15 s at 94 °C, 30 s at 60 °C with −1 °C/cycle, and 3 min at 68 °C, a final PCR step of 30 cycles of 15 s at 94 °C, 30 s at 45 °C, and 3 min at 68 °C with a time increment of +5 s/cycle, and a final elongation step of 7 min at 68 °C. The reaction was then cooled down to 4 °C and nested PCR products were stored at −20 °C.

Analysis of the PCR products was done via a 1% agarose gel electrophoresis at 100 V and obtained nested PCR products were purified using the PCR Cleanup Kit protocol from Abbott Molecular Inc (Des Plaines, IL, USA).

### 2.5. Sequencing and Resistance Profile Analysis

Sanger population sequencing (cut-off value of 15%) on 3130xl ABI PRISM Genetic Analyzer (ThermoFisher Scientific) was performed for the purified nested-PCR products by using a BigDye™ Terminator v3.1 Cycle Sequencing kit (Applied Biosystems Inc., Foster City, CA, USA). The sequencing process was separated in two sets of mix reactions per primer including one for genomic regions of difficult hybridization by the designed sequencing primers (Reaction Mix A) and other for regions of easy hybridization (Reaction Mix B) and performed under the following final concentration conditions: Reaction Mix A (BigDye™ Sequencing Buffer (0.75×), 2 µL of BigDye™ Terminator 3.1 Reaction Mix, 1 µL of primers (5 µM stock solution) RV4, FW6, and RV9 separately per reaction, 1 µL of purified template cDNA, and RNase-Free water to make up a reaction volume of 20 µL) and Reaction Mix B (BigDye™ Sequencing Buffer (0.75×), 1 µL of BigDye™ Terminator 3.1 Reaction Mix, 1 µL of primers (5 µM stock solution) RV2PCR, FW1, FW4, FW5, RV6, 1bRV7, and 1aFW8, separately per reaction, 1 µL of purified template cDNA, and RNase-Free water to make up a reaction volume of 10 µL). Cycling conditions included a denaturation step at 96 °C for 5 min, 35 cycles of 5 s at 94 °C, 10 s at 50 °C, and 4 min at 60 °C, which are followed by a cooldown to 4 °C. A purification of the sequencing reaction products was needed before running on the sequencing equipment and nucleotide sequence chromatograms for each sample were obtained.

The primers nucleotide sequences for each sample were joined in a single contig and edited using ChromasPro^®^ software v1.7.6. (Technelysium Pty Ltd., Brisbane, Australia). Finally, complete nucleotide sequences were converted to FASTA format and analyzed online in Geno2pheno (HCV) v0.92 (Saarbrücken, Germany), which provides the baseline resistance analysis profile for each patient. All sequences obtained in this study were submitted to the REGA HCV database from KU LEUVEN.

### 2.6. Statistical Analysis

Statistical analysis was carried out using IBM^®^ SPSS^®^ Statistics v19 (SPSS Inc., Chicago, IL, USA) and Microsoft Excel (v12.0, Microsoft, Redmond, WA, USA). The association between the presence of NS5 RASs and categorical variables was analyzed through a qui-square test with *p*-values below α = 0.05, which indicates statistical significance.

## 3. Results

### 3.1. Statistical Analysis

Using a confidence level of 95%, statistical analysis indicated that treatment failure was not significantly associated with either the class of NS5 RASs (*p* = 0.549) or the mere non-discriminatory presence of baseline NS5 RASs (*p* = 0.232). However, a statistically significant association was found between treatment failure and the presence of specific baseline NS5 RASs known as Y93C/H (*p* = 0.04) in two out of three recurrent patients.

An additional association between the presence of baseline NS5 RASs and HCV genotype was granted in order to find a connection to other relevant variables that are common in the clinical practice of HCV-infection besides treatment failure. Overall, HCV genotype revealed a strong statistically significant association to the non-discriminatory presence of baseline NS5 RASs (*p* = 0.001) with GT1b being the strongest predictor of this association as well as to the class of NS5 RASs (*p* < 0.001) where GT1b and *NS5B* RASs were the strongest predictors of association. Moreover, the HCV genotype also exhibited a statistically significant association to the presence of specific baseline NS5 RASs known as A92E/T (*p* = 0.007), L159F (*p* < 0.001), C316N (*p* < 0.001), and C451I/Y (*p* = 0.007). GT1b is the strongest predictor of these associations.

### 3.2. Prevalence of NS5 RASs and Treatment Outcome

**Figure 2:**
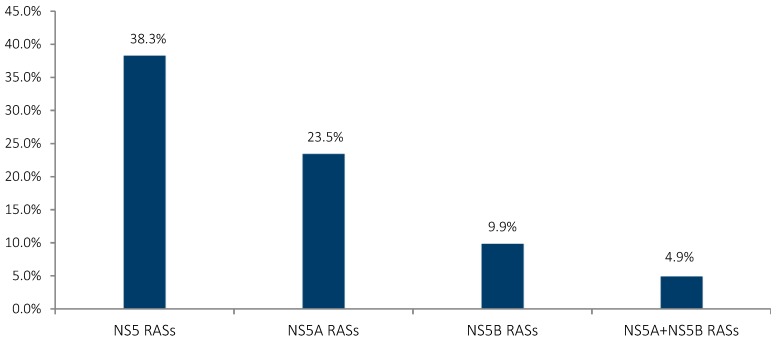
Baseline prevalence of NS5 RASs in all GT1 HCV-infected patients. Substitution analyses were conducted on Sanger sequencing data with a 15% cut-off limit. Dual-class RASs are assigned as *NS5A* + *NS5B* RASs.

Among all genotype 1 infected patients, 38.3% (31/81) presented NS5 RASs at baseline with *NS5A* class RASs showing the highest prevalence. *NS5B* RASs and the combined *NS5A* + *NS5B* RASs followed in the following percentages: 23.5% (19/81), 9.9% (8/81) and 4.9% (4/81), respectively (see Figure 2). However, the prevalence of *NS5A* and *NS5B* RASs would rise up to 28.3% (23/81) and 14.8% (12/81), respectively, when not considering separately the dual-class RASs. Additionally, of the 31 GT1 HCV-infected patients whom presented NS5 RASs at baseline, approximately 58% (18/31) were co-infected with HIV/HCV and disregarded HBV infections.

**Figure 3:**
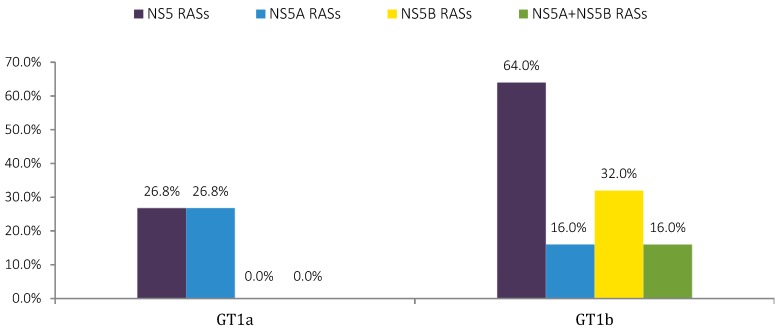
Percentage of HCV-infected patients with NS5 RASs, according to subtype. Substitution analyses were conducted on Sanger sequencing data with a 15% cut-off limit. Dual-class RASs are assigned as *NS5A* + *NS5B* RASs.

Approximately 27% (15/56) of the GT1a infected patients presented NS5 RASs, which was compared to a much higher percentage of NS5 RASs in the GT1b-infected patients that accounted for 64% (16/25) with *NS5B* RASs prevailing at 32% (8/25) followed by *NS5A* and dual-class RASs both at 16% (4/25) (see Figure 3). However, from another point of view, the GT1b patients showed a higher prevalence of *NS5A* RASs than for GT1a, namely 32% (8/25), when not considering separately the dual-class RASs.

Approximately 20% (16/81) of all 81 GT1 infected patients showed at least one RAS that conferred more than 100-fold resistance to ledipasvir (Q30H/R, L31M, and/or Y93C/H) including 21.4% (12/56) and 16% (4/25) of GT1a and GT1b infected patients, respectively. Moreover, dual-class RASs were only observed in 4 GT1b infected patients while combined intra-class RASs were detected in four GT1a patients concerning the *NS5A* region (Q30H + Y93H) and afterward in one and six GT1b patients for *NS5A* (A92T + Y93H) and *NS5B* (L159F + C316N and L159F + C316N + C451Y) genes, respectively.

**Figure 4:**
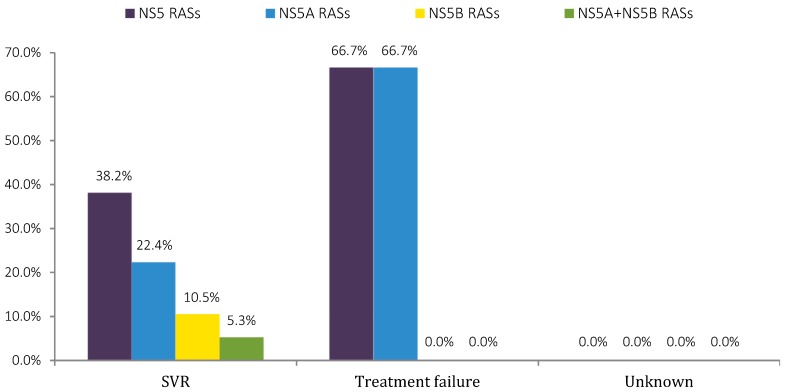
Percentage of HCV infected patients with NS5 RASs, according to treatment outcome status. Substitution analyses were conducted on Sanger sequencing data with a 15% cut-off limit. Dual-class RASs are assigned as *NS5A* + *NS5B* RASs.

Baseline resistance testing of the NS5 coding region showed that the efficacy of SOF and *NS5A* inhibitors, mainly LDV, was not compromised in most patients who showed a natural presence of RASs before treatment initiation since NS5 RASs were present in 38.2% of patients who achieved an SVR with 22.4% (17/76), 10.5% (8/76), and 5.3% (4/76) corresponding to *NS5A*, *NS5B*, and dual-class RASs, respectively (see Figure 4).

Nonetheless, 66.7% (2/3) of the relapsing patients presented *NS5A* class only RASs, as the combined Q30H + Y93H mutations which caused high level resistance to all *NS5A* inhibitors were detected at baseline in one HIV/HCV GT1a co-infected patient who later failed a treatment with SOF + LDV for 12 weeks. However, another relapsing HIV/HCV GT1a co-infected patient who also failed the same therapeutic settings previously described showed no NS5 RASs at baseline. Furthermore, an isolated Y93H mutation was also detected at baseline in one GT1b mono-infected patient experiencing virological relapse.

Even though the Y93H mutation showed a significant effect on treatment outcome (*p* = 0.04) being detected as a dominant substitution for the two patients who did not achieve SVR12 with *NS5A* RASs, most patients with at least one *NS5A* RAS conferring ˃100-fold resistance to ledipasvir still achieved SVR12. Moreover, no RASs were detected in the two GT1a infected patients with an unknown treatment outcome.

Overall, 93.5% (29/31) of patients with baseline NS5 RASs achieved SVR12 of which 54.8% (17/31), 25.8% (8/31), and 12.9% (4/31) corresponded to *NS5A*, *NS5B*, and *NS5A* + *NS5B* class RASs, respectively, when compared to only two patients (6.5%) who experienced virological failure carrying *NS5A* RASs.

### 3.3. Prevalence of NS5A Specific RASs

Major *NS5A* RASs were detected in 23.2% (13/56) of GT1a infected patients (M28V, Q30H/R, L31M, and/or Y93C/H) and in 16% (4/25) of GT1b-infected patients (L31M and Y93H). Yet minor *NS5A* RASs were also detected in 3.6% (2/56) of GT1a patients (H58C/P) and in 20% (5/25) of GT1b patients (L28M, H58S, and A92E/T).

**Figure 5:**
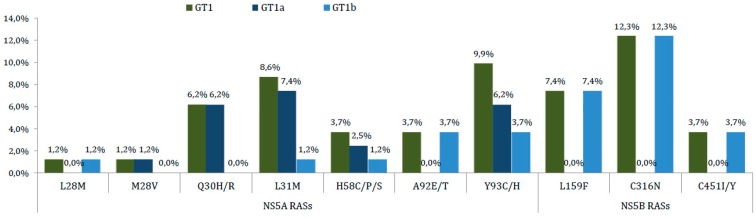
Baseline prevalence of specific NS5 RASs in all 81 HCV-infected patients by HCV subtype. Substitution analyses were conducted on Sanger sequencing data with a 15% cut-off limit. Dual-class RASs are assigned as *NS5A* + *NS5B* RASs.

The most commonly detected *NS5A* RAS was Y93C/H with a baseline prevalence of 9.9% (8/81) in all GT1 infected patients, which was followed by L31M and Q30H/R with a prevalence of 8.6% (7/81) and 6.2% (5/81), respectively (see Figure 5). Moreover, Y93C/H showed a higher prevalence in GT1b patients than in GT1a with percentages of 12% (3/25) vs. 8.9% (5/56), respectively. The baseline Y93C/H mutation largely accounted for 25.8% (8/31) of all patients carrying RASs and was followed by L31M and Q30H/R with percentages of 22.6% (7/31) and 16.1% (5/31), respectively.

### 3.4. Prevalence of NS5B Specific RASs

*NS5B* RASs accounted for 14.8% (12/81) of GT1 HCV-infected patients when not considering separately the dual-class RASs and were only detected in GT1b patients with all achieving SVR12. Furthermore, these were mainly represented by the C316N mutation, which accounted for 40% (10/25) of GT1b-infected patients and showed the highest prevalence among all patients with baseline NS5 RASs with a percentage of 32.3% (10/31). The major S282T mutation causing a high level resistance to sofosbuvir was never detected. However, the baseline L159F RAS that confers a reduced susceptibility to this drug was detected in 24% (6/25) of GT1b-infected patients and in 19.4% (6/31) of all patients carrying NS5 RASs.

## 4. Discussion

HCV sequencing is constantly improving and becoming more widely available for selecting optimal therapy especially in difficult-to-treat patients. In summary, baseline resistance analysis has been shown to be quite useful in selecting increasingly specific regimens for each patient and it helps to achieve a complete viral suppression with high SVR rates countering the progression of the virus in patients with persistent viral loads as well as preventing future therapeutic failures associated with unnecessary high-cost salvage DAAs [25]. Overall, *NS5A* constitutes the most important genomic region considered for the screening of associated RASs. In two studies comparing the effect of baseline *NS5A* RASs on a SOF + LDV based regimen [27,28], the authors found a higher prevalence of baseline RASs for GT1b than for GT1a with percentages of 25% (52/206) and 17.6% (25/142) vs. 13% (40/303) and 7.1% (12/170) for each study respectively, but it is still insignificant on SVR rates except for treatment-experienced patients with decompensated cirrhosis infected with GT1a HCV in which these RASs conferred a high fold resistance (˃100). More recent data from phase II/III studies of 2108 GT1 infected patients evaluating the effects of baseline *NS3*, *NS5A*, and *NS5B* RASs on the response to a SOF + LDV based regimen, showed that up to 16% (338/2108) and 2.5% (43/1692) of patients had detectable *NS5A* and *NS5B* RASs, respectively [29]. This data is further supported from a deep sequencing analysis of baseline *NS5A* RASs in 276 GT1 infected patients, which shows a prevalence of 21% (59/276) of high level *NS5A* RASs conferring resistance to ledipasvir [30]. However, a contemporary study described a much lower prevalence with 11.5% (102/887) of treatment-naive GT1 infected patients showing at least one RAS that conferred ˃100-fold resistance to ledipasvir [29]. Along these lines, all regimens based in *NS5A* inhibitors should include a NI with a high genetic barrier to resistance, currently sofosbuvir, since this combination has been shown to elicit a profound viral suppression [31,32] as well as higher SVR rates when treatment duration is extended to 24 weeks with or without ribavirin. An analysis of 2108 GT1-infected patients who received a regimen of SOF + LDV demonstrated that the reduction in SVR rates appeared to be driven predominantly by patients with *NS5A* RASs, and Y93H was detected as a dominant substitution [29]. Furthermore, another study based on deep sequencing analysis of baseline *NS5A* RASs in 276 and 32 patients infected with GT1 and GT4 HCV, respectively, showed that all patients who failed to achieve SVR12 presented at least one *NS5A* RAS (M28T, Q30H/K/R, L31M/V, and/or Y93C/H) [30].

Regarding *NS5A* specific RASs, a 2015 analysis of more than 3000 GT1 *NS5A* sequences has demonstrated a higher baseline prevalence of the Y93C/H/N mutation especially in GT1b, which was mostly found in South Korea (15.3%) followed by Japan (13.9%) and Spain (13.6%) while, in GT1a, the baseline Q30E/H/R mutation was described as the most commonly detected. The highest prevalence was found in Italy (4.9%), which was followed by France (4.8%) and New Zealand (3.9%) [33]. Moreover, an analysis from the same year of *NS5A* sequences in 132 Japanese GT1b HCV-infected patients established that baseline *NS5A* RASs are common in treatment-naive patients infected with GT1b, which demonstrates an even higher prevalence of the Y93H mutation with a percentage of 25% (32/132) [34].

With regard to the *NS5B* coding region, patients treated with regimens based on *NS5B* polymerase inhibitors, particularly NIs, tend to exhibit a low prevalence of baseline RASs [24], but when detected, the most frequently encountered appear to be L159F, C316N/Y, L320F, V321A, M414T, and S556G [33,35,36,37,38,39,40,41,42,43,44,45]. Dasabuvir, however, possesses a low genetic barrier to resistance since the associated C316N mutation tends to achieve a substantial baseline prevalence of 10% to 36% in GT1b-infected patients [35,46,47,48]. Moreover, an analysis of more than 7800 *NS5A* and *NS5B* sequences from HCV-infected patients across 22 countries showed a prevalence of up to 34% for the L159F mutation in GT1b infected patients [33]. Several studies analyzing the natural presence of *NS5B* RASs equally refute an associated significant impact on SVR12 rates [49,50], which further strengthens this study’s data.

In conclusion, the obtained data supports the usefulness of resistance testing prior to treatment initiation, which prevents relapses associated with the presence of baseline RASs. A statistically significant association was found between treatment failure and the baseline presence of specific NS5 RASs known as Y93C/H (*p* = 0.04). With this in mind, an interesting recommendation to take into account would be to implement a protocol of baseline resistance testing directed only for the clinically-relevant Y93 amino acid position of the *NS5A* gene since all other NS5 RASs, excluding the rare S282T mutation which was not detected, do not seem to have a significant impact on a SOF/LDV based treatment. However, this reduced sampling can constitute a limiting factor since it may underestimate statistical analysis results and lead to overall higher RASs rates when compared to previous studies [24,29,33,34,35,37,43,50,51,52,53]. Furthermore, additional clinical features of the enrolled patients such as RBV use, regimen’s length, fibrosis stage, and degree of portal hypertension were unavailable at the time of this study and response rates over these parameters were unobtainable. These factors are predictors of failure and could have very well enhanced the role of baseline RASs in response to treatment. Along these lines, baseline NS5 RASs seem to have minimal effects on patient responses to sofosbuvir plus *NS5A* inhibitors (LDV, DCV, VEL) based therapies. It is well established through cumulative research data that, when baseline *NS5A* RASs in particular do have effects, they could be largely overcome by extending treatment duration and/or through treatment intensification with the addition of RBV, which drastically reduced the impact of *NS5A* baseline RASs [25,49,54,55,56]. Additionally, the cost-effectiveness of baseline resistance testing was accessed in a recent paper describing how the inquiry of baseline *NS5A* RASs prior to treatment with EBR/GZR is a cost-effective measure resulting in more QALYs (quality-adjusted life years) among GT1a treatment-naive or treatment-experienced patients when compared to a treatment with EBR/GZR without baseline resistance testing. LDV/SOF or 3D based regimens are, therefore, another factor in favor of baseline resistance testing prior to treatment initiation [57].

Lastly, the importance of total adherence to the treatment should always be encouraged and insisted upon in an elucidative way by physicians in order to prevent potential relapses in patients since lack of adherence to treatment is perhaps the main cause of RASs-related treatment failure.

## Figures and Tables

**Table 1:** Demographic baseline characteristics of the HCV-infected patients and treatment outcomes.

DAA-Naive HCV Infected Patients (*n* = 81)	
Mean age, years (range)	49 (23–81)
Male sex, *n* (%)	63 (78%)
Genotype, *n* (%)	
GT1a	56 (69%)
GT1b	25 (31%)
Monoinfection and co-infection profile, *n* (%)	
HCV monoinfected	26 (32%)
HIV/HCV	42 (52%)
HCV/HBV	4 (5%)
HIV/HCV/HBV	9 (11%)
Mean log_10_ HCV RNA, IU/mL (range)	6.22 (4.8–7.4)
HCV RNA, IU/mL, *n* (%)	
<2 million	43 (53%)
2–6 million	30 (37%)
>6 million	8 (10%)
IL28B, *n* (%)	
CC	23 (28%)
CT	47 (58%)
TT	6 (8%)
Unknown	5 (6%)
Outcome after treatment with DAAs, *n* (%)	
SVR12 or SVR24	76 (94%)
Treatment failure	3 (4%)
Unknown	2 (2%)

## Supplementary Materials

The following are available online at http://www.mdpi.com/1999-4915/10/5/223/s1, Table S1: Primers for RT-PCR/cDNA synthesis (outer PCR), nested PCR (inner PCR), and sequencing of HCV NS5 coding region.
