# Supplementary material for: Characterization of NS5A and NS5B Resistance-Associated Substitutions from Genotype 1 Hepatitis C Virus Infected Patients in a Portuguese Cohort
Source: Viruses. 2018 Apr 26;10(5):223. doi: 10.3390/v10050223 (PMC5977216; doi:10.3390/v10050223)
Supplement: Supplementary file 1 [file viruses-10-00223-s001.zip › viruses-297539-supplementary.pdf]

Supplementary Materials:

Table S1. Primers for RT-PCR/cDNA synthesis (outer PCR), nested PCR (inner PCR) and sequencing of HCV NS5 coding region.

Primer name	Purpose	Direction	Primer sequence (5′- 3′)	Location (reference H77) (bp)	GC content	Average Tm
FW1PCR	RT-PCR NS5A-NS5B region	Sense	GTCGTCACBAGYACCTGG	5311 - 5328	62%	56ºC
RV1PCR	RT-PCR NS5A-NS5B region	Antisense	GAGACASGCTGTGATAWATG	9297 - 9316	45%	51ºC
FW2PCR	Nested PCR NS5A-NS5B region / Sequencing NS5A-NS5B region –Back-up option	Sense	GTGGTCATHGTRGGYAGG	5386 - 5403	57%	53ºC
RV2PCR	Nested PCR NS5A-NS5B region / Sequencing NS5A-NS5B region	Antisense	CCCTATTGATYTCACCTGG	9055 - 9073	50%	51ºC
FW1	Sequencing NS5A-NS5B region	Sense	GCHGTGCARTGGATGAA	6118 - 6134	52%	53ºC
RV1	Sequencing NS5A-NS5B region–Back-up option	Antisense	CCRTTYYTGACATGTCC	6493 - 6509	50%	49ºC
FW2	Sequencing NS5A-NS5B region–Back-up option	Sense	TGGMGRCARGAGATG	7093 - 7107	57%	49ºC
RV2	Sequencing NS5A-NS5B region–Back-up option	Antisense	GGRTCRGTRAGCATGGA	6901 - 6917	56%	53ºC
FW3	Sequencing NS5A-NS5B region–Back-up option	Sense	TCYTCYATGCCCCCYCT	7559 - 7574	62%	57ºC
RV3	Sequencing NS5A-NS5B region–Back-up option	Antisense	TTRTTYTCYGACTCMAC	7126 - 7142	41%	46ºC
FW4	Sequencing NS5A-NS5B region	Sense	AARGTCACHTTTGACAG	7816 - 7832	40%	46ºC
RV4	Sequencing NS5A-NS5B region	Antisense	GCBGARTGYGGGGGCGTCAG	7936 - 7955	73%	65ºC
FW5	Sequencing NS5A-NS5B region	Sense	AAGCCAGCTCGYCTYATCGT	8128 - 8147	55%	59ºC
RV5	Sequencing NS5A-NS5B region –Back-up option	Antisense	ACGAGCATBGTGCAGTC	8593 - 8609	60%	55ºC
FW6	Sequencing NS5A-NS5B region	Sense	CTTCACGGAGGCTATGAC	8679 - 8696	56%	52ºC
RV6	Sequencing NS5A-NS5B region	Antisense	GACCADGAYCCGTCRCT	7606 - 7619	61%	55ºC
FW7	Sequencing NS5A-NS5B region–Back-up option	Sense	TYTACCARTGYTGTGAC	8381 - 8397	44%	48ºC
1bRV7	Sequencing GT1b NS5A-NS5B region	Antisense	CATCTCCTGCCGCCACA	7067 - 7083	65%	58ºC
1aFW8	Sequencing GT1a NS5A-NS5B region	Sense	ACACTCGCTGCCVCTGTG	6433 - 6450	65%	60ºC
1bRV8	Sequencing GT1b NS5A-NS5B region –Back-up option	Antisense	AGGTCAAGTGGC TCAATGGA	8987 - 9004	50%	56ºC
1aFW9	Sequencing GT1a NS5A-NS5B region –Back-up option	Sense	CTTCACGGAGGCTATGACC	8636 - 8654	58%	55ºC
RV9	Sequencing NS5A-NS5B region	Antisense	ACGATRAGRCGAGCTGGCTT	8083 - 8102	55%	59ºC
1aFW10	Sequencing GT1a NS5A-NS5B region –Back-up option	Sense	GACAGCAAGACA CACTCC	8816 - 8833	56%	53ºC
1bFW11	Sequencing GT1b NS5A-NS5B region –Back-up option	Sense	AACTCCTGGCTAGGCAACAT	8848 - 8867	50%	56ºC
